# Separation of Adjacent Light Rare Earth Elements Using Silica Gel Modified with Diglycolamic Acid

**DOI:** 10.3390/ma17112648

**Published:** 2024-05-30

**Authors:** Takeshi Ogata, Hirokazu Narita

**Affiliations:** Resource Circulation Technology Research Team, Global Zero Emission Research Center, National Institute of Advanced Industrial Science and Technology (AIST), 16-1, Onogawa, Tsukuba 305-8569, Japan; hirokazu-narita@aist.go.jp

**Keywords:** rare earth elements, separation, adsorbent, adsorption, recycling

## Abstract

The separation of adjacent rare earth elements (REEs) is a challenging issue due to their chemical similarity. We have investigated the separation of adjacent REEs using four types of adsorbents consisting of silica gel modified with diglycolamic acid with different functional groups at the amide position. For all the adsorbents, the adsorption ratio of REEs increased with the increase in atomic number from La to Sm and then became constant for heavy REEs. Among them, EDASiDGA, an adsorbent containing secondary and tertiary amides, showed a high separation factor for Nd/Pr of 2.8. The EDASiDGA-packed column was tested for individual recovery of Pr, Nd, and Sm. After the adsorption of these REEs from 0.10 M HCl, desorption tests were performed with 0.32 and 1.0 M HCl. As a result, Pr and Nd were eluted separately with 0.32 M HCl, and Sm was recovered with 1.0 M HCl. Since the EDASiDGA-packed column showed excellent separation of Pr/Nd/Sm without any chelating agent, it is promising for practical use.

## 1. Introduction

Rare earth elements (REEs) are a group of 17 elements consisting of Sc, Y, and the lanthanides, and they are traditionally divided into two groups: the light rare earth elements (LREEs: La, Ce, Pr, Nd, Sm, and Eu) and the heavy rare earth elements (HREEs: Gd, Tb, Dy, Ho, Er, Tm, Yb, Lu, and Y). REEs are critical elements in a carbon-neutral society due to their use in clean energy technologies, such as wind turbines and electric vehicles; therefore, demand for rare earth magnets whose main components are LREEs such as Nd is expected to grow in the future [1,2,3].

REEs are mostly present as mixtures in primary resources, and therefore, it is necessary to separate each REE from the others for industrial application. Solvent extraction is one of the most common techniques for the separation of REEs because of its excellent selectivity, continuity, and large loading capacity. However, it is difficult to recover REEs individually due to their similar physicochemical properties. For example, acidic organophosphorus extractants such as bis(2-ethylhexyl) hydrogen phosphate (D2EHPA) and mono-2-ethylhexyl (2-ethylhexyl)phosphonate (PC-88A) are typical industrial extractants for the separation of adjacent REEs, which have a large separation factor (*SF*) for REEs, e.g., *SF*s for Nd/Pr by D2EHPA and PC-88A are 1.38 and 1.55, respectively [4]. However, this value of *SF* would not necessarily be cost-effective to obtain high-purity Pr and Nd, respectively. Therefore, the development of separation reagents/separation methods with larger *SF* than conventional ones is completely imperative to make REE refining more efficient.

For the recycling of REEs from secondary resources, it is important to have a technology that allows the separation of small amounts of REEs on demand rather than continuous separation on a large scale as in the case of separation of REEs from primary resources; the adsorption method is suitable for this purpose. The industrial separation method for REEs by adsorption is generally based on the use of ion exchange resins and complexing agents [5,6,7]. However, the presence of chelating agents requires a further purification process that is very costly. Therefore, an adsorbent with a high *SF* can be used to achieve separation with a simple operation. In the basic research phase, many adsorbents have been studied and many reports are available. Examples include polymer-based adsorbents (ion-imprinted polymer-based adsorbents, impregnation of functional groups into adsorbents, etc.), carbon-based adsorbents (graphene oxide, carbon nanotubes, etc.), silica-based adsorbents (silica beads, zeolites, etc.), metal–organic frameworks (MOFs), clay minerals (kaolinite, halloysite, illite, expanded vermiculite, etc.), and biosorbents (cellulose-based adsorbents, chitosan-based adsorbents, etc.) [6,7,8,9,10,11]. Most of these adsorbents are designed to separate only REEs or REEs from other impurities such as Fe or Al. Focusing on the individual separation of REEs, there are several reports on the separation of HREE and LREE, such as the separation of Dy and Nd, and separation is possible [12,13,14,15]. However, there are no reports of effective adsorbents for adjacent REEs, such as Nd and Pr, and classical industrial methods must be used to separate individual REEs by adsorption. We have developed a diglycolamic acid-type adsorbent for the recovery of REEs from unutilized resources [16,17,18], which can selectively recover REEs in the presence of base metals with concentrations more than 1000 times higher [19]. Since this adsorbent shows that the adsorption capacity for LREEs increases linearly with increasing atomic number, it could be applied to the separation of LREEs.

In this study, four types of diglycolamic acid adsorbents were prepared and their separation ability for adjacent REEs was evaluated. In particular, we focused on the separation of Pr, Nd, and Sm, which are used in rare earth magnets.

## 2. Materials and Methods

### 2.1. Reagents

Stock solutions of RE ions were prepared from the following reagents as received from the specified suppliers (with their purity in parentheses): yttrium(III) chloride hexahydrate (99.9%), lanthanum(III) chloride heptahydrate (99.9%), cerium(III) chloride heptahydrate (97.0%), neodymium(III) chloride hexahydrate (97.0%), samarium(III) chloride hexahydrate (99.5%), gadolinium(III) chloride hexahydrate (99.9%), terbium(III) chloride hexahydrate (99.9%), dysprosium(III) chloride hexahydrate (99.5%), holmium(III) chloride hexahydrate (99.9%), erbium(III) chloride hexahydrate (95.0%), ytterbium(III) chloride hexahydrate (99.9%) (Wako Pure Chemical Industries, Tokyo, Japan), scandium(III) chloride hexahydrate (99.9%), praseodymium(III) chloride heptahydrate (99.9%), thulium(III) chloride hydrate (99.9%), lutetium(III) chloride hexahydrate (99.9%) (Strem Chemicals, Newburyport, MA, USA), and europium(III) chloride hexahydrate (96.0%) (Tokyo Chemical Industry, Tokyo, Japan). Adsorbents were prepared from the following reagents as received from the specified suppliers: diglycolic anhydride (Tokyo Chemical Industry, Tokyo, Japan), dichloromethane, and ethanol (Wako Pure Chemical Industries, Tokyo, Japan). We used four kinds of amino-silica gels: 3-aminopropyl silica gel (ASi), which contains a primary amine; 3-(methyleneamino)propyl silica gel (MeASi) and 3-(butyleneamino)propyl silica gel (BuASi), which contain a secondary amine; and 3-(ethylenediamino)propyl silica gel (EDASi), which contains primary and secondary amines. These amino-silica gels, which were purchased from Fuji Silysia, Aichi, Japan, had approximately the same particle size (ca. 100 μm), pore size (ca. 10 nm), specific surface area (ca. 300 m^2^/g), and amount of amino group (ca. 1 mmol/g). All chemicals and solvents used for the synthesis of the adsorbents were used without further purification. Ultrapure water was used for all experiments.

### 2.2. Preparation of Adsorbents

**Figure 1:**
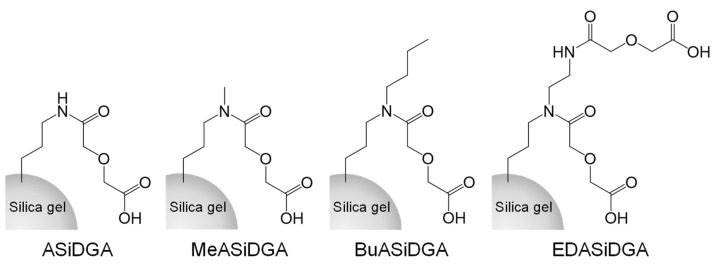
Chemical structures of ASiDGA, MeASiDGA, BuASiDGA, and EDASiDGA.

The adsorbents, which were silica gel-modified with diglycolamic acid, were prepared as described in our previous paper [16,20]. Briefly, diglycolic anhydride and amino-silica gels were added to dichloromethane and allowed to react at 298 K for 3 days, after which the particles were filtered off and washed with dichloromethane, ethanol, and water. The washed particles were dried under vacuum. The adsorbents made from these amino-silica gels are referred to as ASiDGA, MeASiDGA, BuASiDGA, and EDASiDGA (Figure 1), respectively. The results of attenuated total reflection Fourier transform infrared spectroscopy of the prepared adsorbents are shown in the Supplementary Materials.

### 2.3. Procedure of Batch Adsorption Tests

In the batch adsorption experiments, the adsorbent (50 or 100 mg) was added to 5 or 10 mL of a solution containing RE ions (0.1 or 1.0 mM), and the mixture was shaken at 298 K for 3 days. After filtration, the concentration of each ion in the filtrate was determined with an inductively coupled plasma atomic emission spectrometer (ICPE-9000, Shimadzu, Kyoto, Japan) or an inductively coupled plasma mass spectrometer (ICPMS-2030, Shimadzu, Kyoto, Japan), and the adsorbed amount of each ion was calculated. Adsorption ratio, adsorption amount (*Q*), distribution ratio (*K*_d_), and *SF* were obtained from the following formulas. The estimated uncertainty was <5%.

Adsorption ratio [%] = (*C*_0_ − *C*)/*C*_0_ × 100 (1)

*Q* [mmol/g] = (*C*_0_ − *C*) × *V*/*w* (2)

*K*_d_ [L/g] = *Q*/*C* (3)

*SF*_A/B_ = *K*_d,A_/*K*_d,B_ (4)

In the above equations, *C*_0_ is the initial concentration; *C* is the concentration of each metal ion in the filtrate; *V* is the solution volume; and *w* is the weight of the adsorbents.

### 2.4. Procedure of Column Separation

The column test was performed as follows: the adsorbent, EDASiDGA, was packed into a glass column (5 mm inner diameter, 100 mm height). The volume of the adsorbent bed and the dry weight of the adsorbent in the column were 1.96 cm^3^ and 1.06 g, respectively. The column was conditioned with 0.1 M HCl. The adsorption solution ([Pr], [Nd], and [Sm]; 0.1 mM, pH 1.0, 5.9 mL), wash solution (HCl, pH 1.0, 5.9 mL), and desorption solution 1 (HCl, pH 0.50, 29.4 mL) were sequentially passed through the conditioned column at a constant flow rate (space velocity: 3 h^−1^), and finally, the desorption solution 2 (HCl, pH 0.50, 14.7 mL) was passed through the column at a space velocity of 5 h^−1^, and the effluent was collected. The concentration of each element in the aqueous solution was measured for each fraction using the ICP atomic emission spectrometer. The column experiment was performed at room temperature (291 ± 2 K).

## 3. Results and Discussion

### 3.1. Batch Adsorption Tests of REEs

**Figure 2:**
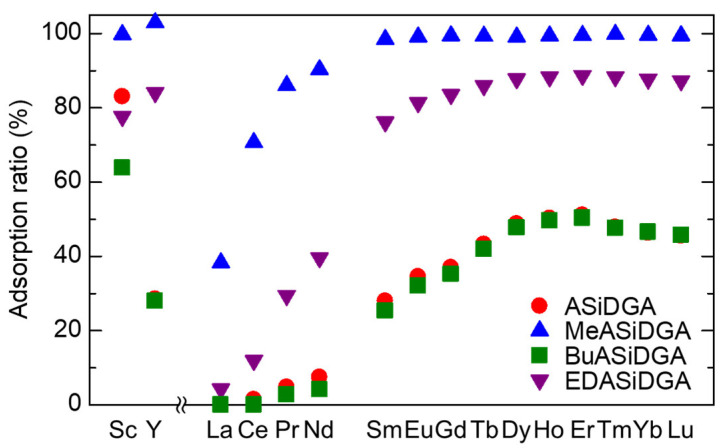
Adsorption ratios of REEs onto ASiDGA, MeASiDGA, BuASiDGA, and EDASiDGA. Initial conc. of each RE ion, 0.10 mM; initial pH, 1.0; pH adjustment, HCl, *V*, 5 mL; adsorbent, 100 mg.

Figure 2 shows the results of adsorption tests of mixtures containing 16 REEs by the four prepared adsorbents. The adsorption trends were similar for four types of diglycolamic acid adsorbents: for the LREEs, the higher the atomic number, the higher the adsorption ratio; for the HREEs and Sc, each ion had a uniformly high adsorption ratio; and the adsorption ratio of Y was similar to that of Sm. These results for lanthanides show the same trend as those obtained by solvent extraction with diglycolamic acid extractant [21,22]. The La^3+^ complex with *N*-butyldiglycolamic acid has three diglycolamic acid ligands that coordinate to the La^3+^ ion in a tridentate fashion through the amide, ether, and carboxyl oxygen atoms [22]. Accordingly, it is assumed that the adsorption of REEs by diglycolamic acid adsorbents has a similar coordination structure. The magnitude of the adsorption ratios of the REEs showed a difference among the adsorbents. The lowest magnitude was observed for ASiDGA and BuASiDGA. For ASiDGA, this was due to the low coordination ability of the secondary amides when forming a hydrogen bond between the oxygen atom and the N–H in the amide group, since the RE ion can coordinate with three oxygen donor atoms in the carboxyl, ether, and amide groups [20]. MeASiDGA, a tertiary amide, had the highest adsorption ratios for REEs, in contrast to BuASiDGA, also a tertiary amide. The low adsorption ratio of BuASiDGA was probably due to the hydrophobic butyl side chain of the amide group, which reduced the contact efficiency between the adsorbent site and the aqueous solution. For EDASiDGA, large differences in adsorption ratios between adjacent REEs were observed for LREEs.

**Figure 3:**
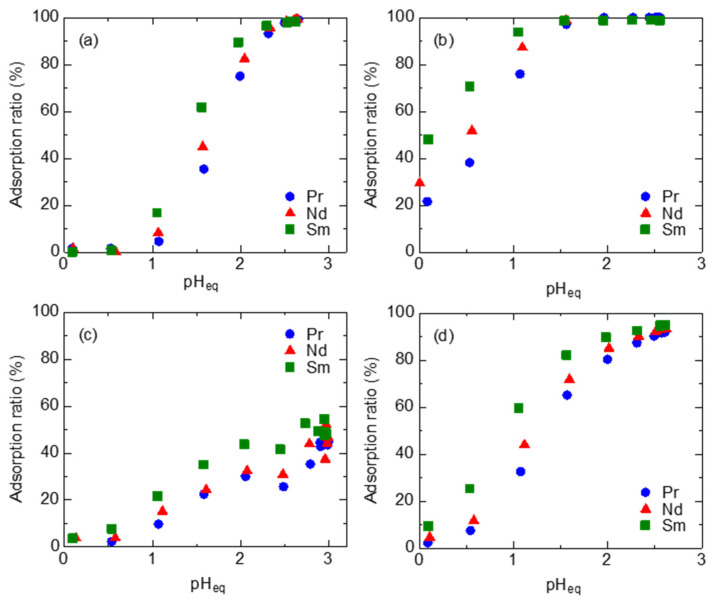
Equilibrium pH dependence of adsorption ratios of Pr, Nd, and Sm by (**a**) ASiDGA, (**b**) MeASiDGA, (**c**) BuASiDGA, and (**d**) EDASiDGA. Initial conc. of Pr, Nd, and Sm ion, 1.0 mM; initial pH, 0–3.0; pH adjustment, HCl, V, 5 mL; adsorbent, 50 mg.

Figure 3 shows the pH dependence of the adsorption ratios using single-component systems of Pr, Nd, and Sm, respectively. The adsorption ratio increased with increasing pH for each adsorbent. This result confirms that the deprotonation of the carboxylic acid group in diglycolamic acid occurs during the REE adsorption. BuASiDGA showed lower adsorption ratios than the other adsorbents at all pHs tested. ASiDGA adsorbed LREEs at pH 1 or higher. On the other hand, MeASiDGA adsorbed LREEs at a low pH. It was found that the difference in the adsorption ratios of Pr, Nd, and Sm on EDASiDGA becomes large at pH 0.50–1.5. These results indicate that EDASiDGA has a high potential for Pr/Nd/Sm separation.

**Figure 4:**
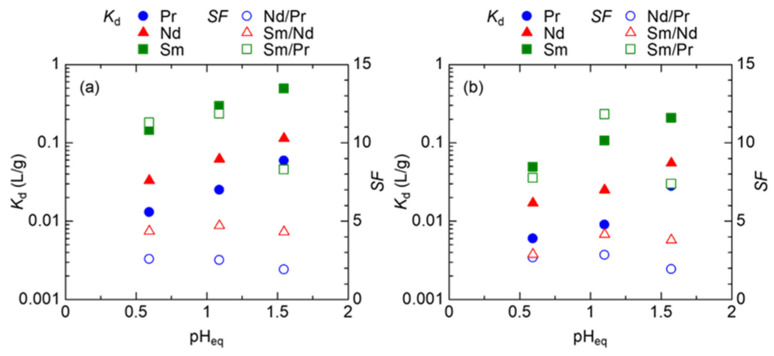
Distribution ratios and separation factors by (**a**) MeASiDGA and (**b**) EDASiDGA from the solution with coexisting Pr, Nd, and Sm. Initial conc. of Pr, Nd, and Sm ions, 1.0 mM; initial pH, 0.50–1.5; pH adjustment, HCl; V, 10 mL; adsorbent, 100 mg.

The *SF* for Nd/Pr, Sm/Nd, and Sm/Pr were investigated by using a mixed solution of Pr, Nd, and Sm. We performed adsorption tests on MeASiDGA and EDASiDGA, which are expected to have good separation of LREEs. The adsorption results are shown in Figure 4, and the *SF*s for Nd/Pr, Sm/Nd, and Sm/Pr are summarized in Table 1. As with the single-component systems, the *K*_d_ of Pr, Nd, and Sm increased with increasing pH of the mixed solution. It was confirmed that at pH 1.5, the *K*_d_ of Pr increased more than that of Nd and Sm, and as a result, the *SF*s for Nd/Pr and Sm/Pr became smaller. Therefore, the pH range of 0.59 to 1.1 is considered the optimal condition where high separation performance can be expected. Table 2 shows the *SF*s for Nd/Pr, Sm/Nd, and Sm/Pr values obtained by a well-known industrial adsorption method using ion exchange resin with aminocarboxylic acid chelating agents; here, the *SF*s for Nd/Pr, Sm/Nd, and Sm/Pr were calculated based on the stability constants of REEs and the aminocarboxylic acid chelating agents because there is almost no difference in the stability constants of REEs in the REE ion exchange resin system [5,6,7]. A comparison of the *SF*s showed that the prepared adsorbents were superior in almost all *SF* values in the pH range of 0.59 to 1.1. Especially for Nd and Pr, EDASiDGA showed a maximum *SF*_Nd/Pr_ of 2.8.

### 3.2. Column Tests with EDASiDGA

**Figure 5:**
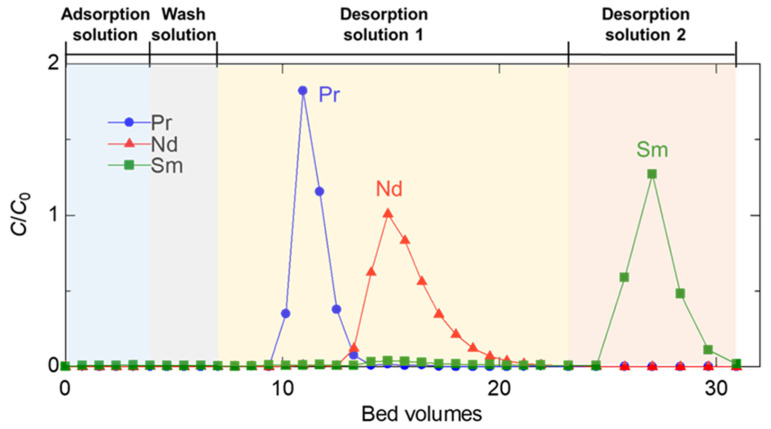
Separation test of Pr, Nd, and Sm using EDASiDGA-packed column. Adsorption solution, ([Pr], [Nd], and [Sm]; 0.10 mM, pH 1.0); wash solution (HCl, pH 1.0); desorption solution 1 (HCl, pH 0.50); desorption solution 2 (HCl, 1.0 M).

A separation test of LREEs was performed using a column packed with EDASiDGA, which has a high *SF* value for LREEs. The adsorption solution (pH 1.0 solution containing Pr, Nd, and Sm), wash solution (HCl, pH 1.0), desorption solution 1 (HCl, pH 0.50), and desorption solution 2 (HCl, 1.0 M) were sequentially passed through the column. Figure 5 shows the ratio of the concentration of each ion in the effluent (*C*) to its initial concentration (*C*_0_) versus the bed volume (BV). When the adsorption test solution containing Pr, Nd, and Sm was passed through the EDASiDGA-packed column, all LREEs were captured on the column, i.e., *C*/*C*_0_ = 0. The adsorbed LREEs were not eluted by the wash solution. Using the desorption solution 1, Pr was desorbed first, followed by Nd. Then, the adsorbed Sm was eluted out by the desorption solution 2. Accordingly, Pr, Nd, and Sm were recovered individually by using the EDASiDGA-packed column. Unlike the conventional adsorption system using a large amount of chelating agent, this column can precisely separate Pr, Nd, and Sm from each other simply by varying the HCl concentration in a narrow range of 0.10–1.0 M without any chelating agent. This is due to the high *SF* between adjacent REEs by EDASiDGA, an adsorbent, and the easy desorption of the adsorbed REEs with about 1.0 M HCl. These properties support that the EDASiDGA-packed column is promising for practical separation of Pr, Nd, and Sm.

## 4. Conclusions

We prepared four types of adsorbents with different groups attached to the nitrogen atom of the amide bond of diglycolamic acid and investigated the separation of adjacent REEs by batch and column adsorption tests. Regarding the relationship between the atomic number and adsorption ratio of REE, all the adsorbents displayed a similar trend; the adsorption ratio increased from La to Sm and then became almost constant for HREEs. However, the difference in the degree of *SF* and adsorption ratio was obviously seen for each adsorbent. Adsorbents with tertiary amides were more adsorptive to REEs than those with secondary amides; the shorter the carbon chain attached to the nitrogen atom of the amide, the higher the adsorption performance. EDASiDGA, an adsorbent with secondary and tertiary amides, showed a maximum *SF*_Nd/Pr_ of 2.8. The separation of Pr, Nd, and Sm was tested using a small column packed with EDASiDGA. Each element was recovered quantitatively with high selectivity. Unlike the conventional adsorption technique, the separation process using EDASiDGA does not require the addition of chelating agents, which would make REE refining smaller and more cost-effective.

## Figures and Tables

**Table 1:** Separation factors for Nd/Pr, Sm/Nd, and Sm/Pr by MeASiDGA and EDASiDGA.

pH_eq_	MeASiDGA			EDASiDGA		
	Nd/Pr	Sm/Nd	Sm/Pr	Nd/Pr	Sm/Nd	Sm/Pr
0.59	2.6	4.4	11	2.7	2.9	7.8
1.10	2.5	4.7	12	2.8	4.2	12
1.57	1.9	4.3	8.3	1.9	3.8	7.4

**Table 2:** Separation factors for Nd/Pr, Sm/Nd, and Sm/Pr calculated from the stability constants (*K*_1_) of REEs and aminocarboxylic acid chelating agents.

	log *K*_1_			*SF*		
	Pr	Nd	Sm	Nd/Pr	Sm/Nd	Sm/Pr
IDA ^a^	3.84	4.07	4.57	1.70	3.16	5.37
NTA ^b^	9.69	9.87	10.21	1.51	2.19	3.31
HEDTA ^c^	14.17	14.47	14.85	2.00	2.40	4.79
EDTA ^d^	16.14	16.31	16.76	1.48	2.82	4.17

a: iminodiacetic acid [23], b: nitrilotriacetic acid [24], c: *N*′-(2-hydroxyethyl)ethylenediamine-*N*,*N*,*N*′-triacetic acid [25], d: ethylenediamine-*N*,*N*,*N*′,*N*′-tetraacetic acid [26].

## Data Availability

The original contributions presented in the study are included in the article/Supplementary Materials, further inquiries can be directed to the corresponding author.

## Supplementary Materials

The following supporting information can be downloaded at: https://www.mdpi.com/article/10.3390/ma17112648/s1, Figure S1: ATR FT-IR spectra of unmodified amino-silica gels and synthesized adsorbents; Table S1: Peaks (cm^−1^) in ATR FT-IR spectra of synthesized adsorbents.

## References

[1] Morimoto S., Kuroki H., Narita H., Ishigaki A. (2021). Scenario assessment of neodymium recycling in Japan based on substance flow analysis and future demand forecast. J. Mater. Cycles Waste Manag..

[2] Yao T., Geng Y., Sarkis J., Xiao S., Gao Z. (2021). Dynamic neodymium stocks and flows analysis in China. Resour. Conserv. Recycl..

[3] Heim J.W., Vander Wal R.L. (2023). NdFeB Permanent Magnet Uses, Projected Growth Rates and Nd Plus Dy Demands across End-Use Sectors through 2050: A Review. Minerals.

[4] Jha M.K., Kumari A., Panda R., Kumar J.R., Yoo K., Lee J.Y. (2016). Review on hydrometallurgical recovery of rare earth metals. Hydrometallurgy.

[5] Gupta C.K., Krishnamurthy N. (2005). Extractive Metallurgy of Rare Earths.

[6] Ouardi Y.E., Virolainen S., Mouele E.S.M., Laatikainen M., Repo E., Laatikainen K. (2023). The recent progress of ion exchange for the separation of rare earths from secondary resources—A review. Hydrometallurgy.

[7] Pathapati S.V.S.H., Free M.L., Sarswat P.K. (2023). A Comparative Study on Recent Developments for Individual Rare Earth Elements Separation. Processes.

[8] Salfate G., Sánchez J. (2022). Rare Earth Elements Uptake by Synthetic Polymeric and Cellulose-Based Materials: A Review. Polymers.

[9] Brião G.V., Silva M.G., Vieira M.G.A. (2022). Adsorption potential for the concentration and recovery of rare earth metals from NdFeB magnet scrap in the hydrometallurgical route: A review in a circular economy approach. J. Clean. Prod..

[10] Danouche M., Bounaga A., Oulkhir A., Boulif R., Zeroual Y., Benhida R., Lyamlouli K. (2024). Advances in bio/chemical approaches for sustainable recycling and recovery of rare earth elements from secondary resources. Sci. Total Environ..

[11] Bishop B.A., Alam M.S., Flynn S.L., Chen N., Hao W., Shivakumar K.R., Swaren L., Rueda D.G., Konhauser K.O., Alessi D.S. (2024). Rare Earth Element Adsorption to Clay Minerals: Mechanistic Insights and Implications for Recovery from Secondary Sources. Environ. Sci. Technol..

[12] Roosen J., Spooren J., Binnemans K. (2014). Adsorption performance of functionalized chitosan-silica hybrid materials toward rare earths. J. Mater. Chem. A.

[13] Liu E., Xu X., Zheng X., Zhang F., Liu E., Li C. (2017). An ion imprinted macroporous chitosan membrane for efficiently selective adsorption of dysprosium. Sep. Purif. Technol..

[14] Nishihama S., Harano T., Yoshizuka K. (2018). Silica-based solvent impregnated adsorbents for separation of rare earth metals. Sep. Sci. Technol..

[15] Hoshina H., Chen J., Amada H., Seko N. (2020). Chain entanglement of 2-ethylhexyl hydrogen-2-ethylhexylphosphonate into methacrylate-grafted nonwoven fabrics for applications in separation and recovery of Dy(III) and Nd(III) from aqueous solution. Polymers.

[16] Ogata T., Narita H., Tanaka M. (2014). Immobilization of diglycol amic acid on silica gel for selective recovery of rare earth elements. Chem. Lett..

[17] Ogata T., Narita H., Tanaka M. (2015). Adsorption behavior of rare earth elements on silica gel modified with diglycol amic acid. Hydrometallurgy.

[18] Shinozaki T., Ogata T., Kakinuma R., Narita H., Tokoro C., Tanaka M. (2018). Preparation of polymeric adsorbents bearing diglycolamic acid ligands for rare earth elements. Ind. Eng. Chem. Res..

[19] Ogata T., Narita H., Tanaka M. (2015). Rapid and selective recovery of heavy rare earths by using an adsorbent with diglycol amic acid group. Hydrometallurgy.

[20] Ogata T., Narita H., Tanaka M. (2016). Adsorption mechanism of rare earth elements by adsorbents with diglycolamic acid ligands. Hydrometallurgy.

[21] Naganawa H., Shimojo K., Mitamura H., Sugo Y., Noro J., Goto M. (2007). A new “green” extractant of the diglycol amic acid type for lanthanides. Solvent Extr. Res. Dev..

[22] Shimojo K., Fujiwara I., Fujisawa K., Okamura H., Sugita T., Oshima T., Baba Y., Naganawa H. (2016). Extraction behavior of rare-earth elements using a mono-alkylated diglycolamic acid extractant. Solvent Extr. Res. Dev..

[23] Thompson L.C., Loraas J.A. (1963). Complexes of the rare earths. III. Mixed complexes with N-hydroxyethylethylenediaminetriacetic acid. Inorg. Chem..

[24] Schijf J., Byrne R.H. (2021). Speciation of yttrium and the rare earth elements in seawater: Review of a 20-year analytical journey. Chem. Geol..

[25] Gritmon T.F., Goedken M.P., Choppin G.R. (1977). The complexation of lanthanides by aminocarboxylate ligands—I. Stability constants. J. Inorg. Nucl. Chem..

[26] Suzuki Y., Yokoi S., Katoh M., Minato M., Takizawa N., McCarthy G.J., Rhyne J.J., Silber H.B. (1980). Stability constants of rare-earth complexes with some organic ligands. The Rare Earths in Modern Science and Technology.

