# Supplementary material for: Separation of Adjacent Light Rare Earth Elements Using Silica Gel Modified with Diglycolamic Acid
Source: Materials (Basel). 2024 May 30;17(11):2648. doi: 10.3390/ma17112648 (PMC11173636; doi:10.3390/ma17112648)
Supplement: Supplementary file 1 [file materials-17-02648-s001.zip › materials-3007508-supplementary.pdf]

FT-IR spectra of the prepared adsorbents

Attenuated total reflection Fourier transform infrared (ATR FT-IR) spectroscopy was carried out to determine the functional groups in perpetrated adsorbents with a FT-IR spectrometer (PerkinElmer, Spectrum 100). Before the measurement, the samples were pulverized into powder.

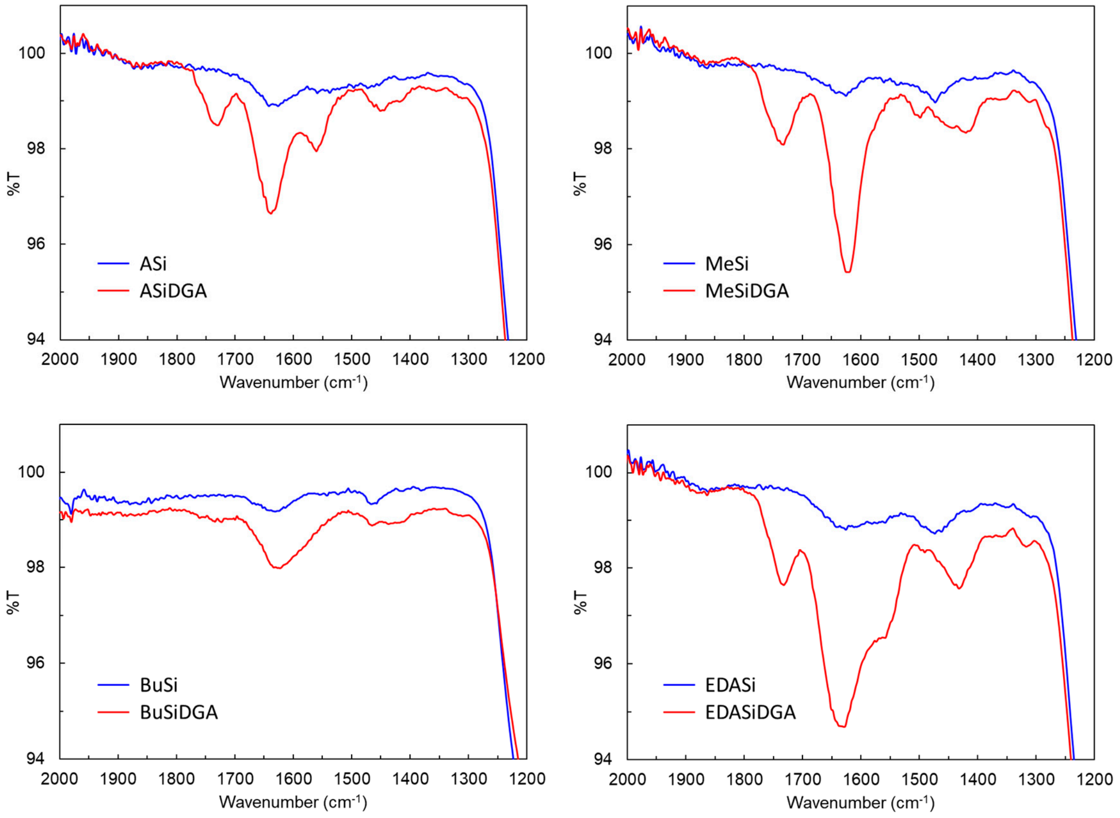

Figure S1. ATR FT-IR spectra of unmodified amino-silica gels and synthesized adsorbents.

Table S1. Peaks (cm^{-1}) in ATR FT-IR spectra of synthesized adsorbents.

Sample	Carboxylic acid	Amide	
	C=O stretching	C=O stretching	N–H bending
ASiDGA	1730	1639	1561
MeSiDGA	1733	1625	—[a]
BuSiDGA	1734	1624	—[a]
EDASiDGA	1732	1638	1566

[a]N–H bending peaks were not observed for MeSiDGA and BuSiDGA, because they are tertiary amide compounds.
